# Emotional and Affective Temperaments in Smoking Candidates for Bariatric Surgery

**DOI:** 10.1371/journal.pone.0150722

**Published:** 2016-03-17

**Authors:** Karin Daniele Mombach, Cesar Luis de Souza Brito, Alexandre Vontobel Padoin, Daniela Schaan Casagrande, Claudio Cora Mottin

**Affiliations:** 1 Post-Graduation Program in Medicine and Health Science, Clinical Sugery, Faculty of Medicine Pontíficia Universidade Católica do Rio Grande do Sul, Porto Alegre, Brazil; 2 Obesity and Metabolic Syndrome Center, Hospital São Lucas da Pontifícia Universidade Católica do Rio Grande do Sul, Porto Alegre, Brazil; 3 Faculty of Medicine, Hospital São Lucas da Pontíficia Universidade Católica do Rio Grande do Sul, Porto Alegre, Brazil; University of Texas Health Science Center at San Antonio, UNITED STATES

## Abstract

**Introduction:**

The prevalence of smoking habits in severe obesity is higher than in the general population. There is some evidence that smokers have different temperaments compared to non-smokers. The aim of this study is to evaluate the associations between smoking status (smokers, ex-smokers and non-smokers) and temperament characteristics in bariatric surgery candidates.

**Methods:**

We analyzed data on temperament of 420 bariatric surgery candidates, as assessed by the AFECTS scale, in an exploratory cross-sectional survey of bariatric surgery candidates who have been grouped into smokers, ex-smokers and non-smokers.

**Results:**

We detected significant statistical differences in temperament related to the smoking status in this population after controlling the current use of psychiatric medication. Smokers had higher anxiety and lower control than non-smokers. Ex-smokers with BMI >50 kg/m^2^ presented higher coping and control characteristics than smokers.

**Conclusions:**

Smoking in bariatric surgery candidates was associated with lower control and higher anxious temperament, when controlled by current use of psychiatric medication. Smokers with BMI >50 kg/m^2^ presented lower coping and control than ex-smokers. Assessment of temperament in bariatric surgery candidates may help in decisions about smoking cessation treatment and prevention of smoking relapse after surgery.

## Introduction

Obesity and tobacco use are major public health problems and are incremental health risk factors. [1–3] The combination of the two disorders increases the risk of mortality. [4] On the basis of previous research, [5–10] the prevalence of smoking in obese patients, prior to occurrence of bariatric surgery, ranges from 16 to 38%. The prevalence of tobacco use in this population is such a significant problem that they have a 2-fold increased risk to be a smoker when compared to people who present normal body mass indexes (BMI). [7]

Temperament, one of the components of personality traits, is related to affective, mood and behavior aspects of human beings. It is relatively stable throughout a life span and it is strongly determined by genetic factors. [11,12] Many investigations have performed expanded studies of personality and temperament and have demonstrated that some temperament traits are associated with obesity [13–15] and smoking. [16–18] We consider that certain temperament traits may also be associated to specific characteristics of bariatric surgery subgroups that might be associate to non-adaptive behavioral expression of this traits influencing difficult on treatment adherence. These characteristics may lead to the elaboration of specific approaches to particular therapeutic interventions in order to promote a higher adhesion to the proposed treatments as well as the achievement of more qualified bariatric results.

There are different models to assess the characteristics of personality and temperament. The main models may be divided into two groups. In the first group, where the McCrae & Costa’s Big Five, [19] and Cloninger, [11] are the most representative, and known models which include Gray, [20] consider temperament under the light of an approach which is closely connected to emotional and behavior systems. The second group presents a more synthetic view, amongst them Eysenck [21] with his three dimensions approach (extraversion, neuroticism and psychoticism) and Akiskal [22] with the model of five affective temperaments (cyclothymic, manic, irritable, depressive, hyperthymic types which represent premorbid traits or constitutional peculiarities). These five affective temperaments are considered the predisposition base for the development of mood disorders (related trait) and present diverse distributions according to the type of mood disorder (for instance, more hyperthymic and irritable traits in bipolar disorder type II).

Lara et al. [23,24] proposed a synthetic and integrative approach to temperament called Fear and Anger model. This model integrates the concepts of emotional temperament which have been previously described by Cloninger et al., [11] along with the concepts of affective temperaments, as proposed by Akiskal et al. [22,25] Recently, Lara et al. [26] developed the Affective and Emotional Composite Temperament (AFECT) model and they also have validated the AFECT scale. The concept of temperament has traditionally involved basic emotions and Lara and his colleagues added regulatory mechanisms (control) to this concept, as well as sensitivity to environmental events and coping skills as being crucial for emotional self-regulation.

In respect to tobacco, several factors can contribute to its use or dependence, such as genetic factors, [27,28] characteristics of individual personality, [29,30] psychiatric disorders [31–33] and environmental influences. [34,35] A history of smoking has been shown to increase the risk of developing postoperative complications (marginal ulcerations, strictures, fistula, venous thromboembolism and infections). [36] The risk of death is twice as likely for active tobacco users. [5]

The latest evidence-based bariatric surgery guidelines recommend to abstain from and stop smoking at least six weeks prior to bariatric surgery, [37] but there are no recommendations about the smoking postoperatively. We don’t find any researches concerning smoking in the post bariatric surgery, that can denote that the professionals who work with bariatric patients have concerns about weight loss and about alcohol abuse after surgery but seem to have few concerns or treatment strategy to treat dependence of tobacco. The clinical impact of smoking and this relation with temperament is because the treatment and prevention strategies (pharmacological and behavior) need to be individualized based on the disadaptive temperament traits of patients.

The objective of this study was to examine the associations between smoking status (smokers, ex-smokers and non-smokers) and temperaments as assessed by AFECTS questionnaire.

Based on previous studies, [16,17,38] our initial hypothesis was that smoking in severe obese individuals would be associated with low drive, low inhibition, low control and cyclothymic, irritable and dysphoric affective temperaments.

## Materials and Methods

### Participants and Procedures

We performed a cross-sectional study with patients who were undergoing their presurgical psychological evaluation. All medical records from patients who were seen at the outpatient clinic of a tertiary referral center for obese (Centro da Obesidade e Síndrome Metabólica, Hospital São Lucas da Pontifícia Universidade Católica do Rio Grande do Sul [Obesity and Metabolic Syndrome Center: COM HSL-PUCRS]) between June 2011 and November 2012 were reviewed, and the patient records was anonymized and de-identified prior to analysis. This study was approved by the Institutional Review Board of the Hospital São Lucas da Pontifícia Universidade Católica do Rio Grande do Sul (PUCRS) in Porto Alegre, Brazil (protocol 11/05563). The inclusion criteria were being 18–65 years of age and presenting BMI ≥ 35 kg/m^2^. The exclusion criteria were patients with cognitive impairment, problems in filling out the questionnaire and presence of psychopathology without any control, such as symptoms of psychosis, absence of control on impulses, aggressiveness, abuse of alcohol and drugs. Psychiatric evaluation was carried out by a psychiatrist using a systematic inquiry protocol which listed all relevant psychiatric diseases on Axis I (DSM-IV-TR): mood disorders (major depressive disorder, bipolar disorder, postpartum depression), eating disorders (anorexia nervosa, bulimia nervosa, binge-eating disorder, eating disorders not otherwise specified), substance use disorder, anxiety disorder, insomnia, attention deficit hyperactivity disorder). The questionnaire (AFECTS) was answered by the patients.

We tested the hypotheses through samples without psychiatric medication at the time of the psychiatric interview. This methodology was proposed in order to minimize potential confounding effects. Medicaments such as antidepressants may provoke mood-switching in hyperthymic, cyclothymic or euphoric individuals, [26] and others medications can interfere with impulsivity or control (such as anticonvulsants). We also tested a subgroup with severe intensity of obesity (BMI>50 kg/m^2^).

### Measures

The AFECTS [26] is a self-report questionnaire and is composed of separate emotional and affective sections.

### The Emotional Section

The emotional section is a 7-point bipolar scale with 48 items, divided into 6 dimensions of 8 questions. These six dimensions are: Volition, Anger, Inhibition, Sensitivity, Coping and Control. The total score of each dimension is the sum of scores from 1 to 7 for each question, so their scores range from 8 to 56.

The AFECTS was validated and showed good psychometric properties in Brazilian Internet Study on Temperament and Psychiatry (BRAINSTEP). [26] In this study, the Cronbach's alpha coefficients indicated acceptable to excellent levels of homogeneity for all AFECTS dimensions: Sensitivity, 0.87; Control, 0.89; Anger, 0.88; Volition, 0.91; Coping, 0.87; Inhibition, 0.75 (Fear, 0.65; Caution, 0.76). Cronbach's alpha coefficients in the present study: Sensitivity, 0.87; Control, 0.76; Anger, 0.88; Volition, 0.89; Coping, 0.79; Inhibition, 0.61 (Fear, 0.64; Caution, 0.77).

### The Affective Section

The Affective section corresponds to question 2 and 3 of the scale AFECTS. [26] this section comprises twelve short descriptions of the putative affective temperaments presented with a 5-item Likert scale, from ‘nothing like me’ to ‘exactly like me’ (rated 1 to 5): depressive, anxious, apathetic, obsessive, cyclothymic, dysphoric, volatile, euthymic, irritable, disinhibited, hyperthymic and euphoric.

Regarding the affective section in the present study we applied the Spearman correlation coefficient and we found similar results to the ones that had been found on BRAINSTEP. [26]

### Assessment of Cigarette Smoking

The subjects were grouped according to their smoking status proposed by Chatkin et al. [7] Current smokers were those who had smoked ≥ 100 cigarettes throughout their lifetime and were still smoking on a daily basis or smoking on most days. Non-smokers were those who had never smoked or smoked < 100 cigarettes in their lifetime and currently were not smoking. Ex-smokers were those who smoked > 100 cigarettes in their lifetime, but had not smoked for >90 days. This later criterion was included by the authors to classify the smoking group that due to the bariatric protocol needed to stop smoking to be submitted to bariatric surgery.

### Statistical Analyses

Demographic variables were analyzed with ANOVA followed by Tukey's test as post hoc for symmetrically distributed data and the chi-square test for categorical data. Data with asymmetric distribution were analyzed by the Kruskal-Wallis test. The scores of emotional and affective dimensions were compared between categories of smoker status (smoker, ex-smoker and non-smoker) with ANOVA followed by Tukey's test as post hoc. We used multivariate linear regression to adjust the association between smoker status and different dimensions of AFECTS for sex and age one some temperaments, such as harm avoidance and fear are higher in women than men and novelty seeking, volition and anger tend to decrease with age. [39,40] P≤0.05 was considered statistically significant. SPSS 18.0 software was used for all the analyses that have been performed.

## Results

The sample consisted of 420 bariatric surgery candidates in this cross-sectional study. Most were female (74.5%) and Caucasian (92.9%) and the mean BMI was 45.9±7.6 kg/m^2^. There were no statistically significant differences between these demographic characteristics and the smoking status sub-groups: smokers (P = 0.404), ex-smokers (P = 0.719) and non-smokers (P = 0.836). The mean preoperative age was 36.7±10.0 years old, and smokers and non-smokers had a lower mean age than ex-smokers (P<0.001). The demographic and clinical characteristics are shown in Table 1.

**Table 1:** Clinical and demographic characteristics of the subjects according to smoking status (n = 420).

Characteristic	Smokers (n = 48)	Ex-smokers (n = 98)	Non-smokers (n = 274)	P
Age (years)	34.5 ± 8.2^a^^,^^b^	39.9± 10.2^b^	36.0 ± 10.0^a^	<0.001
Sex (female) (%)	36 (75.0)	68 (69.4)	209 (76.3)	0.404
Caucasian (%)	44 (91.7)	90 (94.7)	247 (92.9)	0.719
Weight (kg)	125.9 ± 22.0	127.3 ± 24,6	125.5 ± 24.7	0.836
Body mass index (kg/m^2^)	45.1 ± 7.1	46.6 ± 7.8	45.8 ± 7.7	0.530
Any psychiatric disorder diagnosis (%)	26(54.3) ^a^	53(54.1) ^a^	113(41.2) ^b^	0.042
Any current psychiatric medication(%)	13(27.1)	24(24.5)	58(21.2)	0.596

Categorical variables are described by frequency and percentage. Quantitative variables are described by mean and standard deviation for symmetric data or median (P25-P75) for asymmetric data. P for categorical variables was obtained by chi-square test, quantitative symmetric data by ANOVA followed by Tukey’s test, and asymmetric data by the Kruskal-Wallis test.

^a,b^ Different letters represent statistically different values, and same letters represent values statistically equal.

When observing the global results of the smoking status in bariatric surgery candidates, even at the instance of adjustments for sex and age, we had no statistically significant difference between the scores of AFECTS (see Table 2).

**Table 2:** Characteristics of emotional and affective temperaments (AFECTS) of the subjects studied (n = 420).

Characteristic	Smokers (n = 48)	Ex-smokers (n = 98)	No smokers (n = 274)	P
VOLITION	39.31±11.56	38.7±10.36	39.61±10.2	0.762
ANGER	31.23±10.31	27.49±10.76	29.11±10.90	0.138
INHIBITION	29.02±5.81	27.72±6.58	27.39±6.46	0.267
SENSIVITY	31.02±10.03	32.54±10.25	31.46±9.88	0.588
COPING	43.81±10.13	45.76±8.86	45.21±10.51	0.550
CONTROL	40.71±10.65	43.72±8.59	43.97±9.49	0.086
Depressive	1(1–3)	1(1–3)	1(1–3)	0.780
Anxious	3(2–4)	3(1–4)	3(1–4)	0.191
Apathetic	2(1–3)	1(1–3)	1(1–2)	0.121
Obsessive	3(2.25–4)	4(3–5)	4(2–5)	0.310
Cyclothymic	3(1–4)	2(1–3.25)	2(1–4)	0.371
Dysphoric	3(2–4)	3(1–4)	2(1–4)	0.098
Volatile	1(1–3)	1(1–2)	1(1–2)	0.140
Euthymic	4(2–5)	4(3–5)	4(3–5)	0.585
Irritable	4(3–4,75)	3(2.75–4)	4(3–5)	0.123
Disinhibited	2(1–4)	2(1–3)	2(1–4)	0.675
Hyperthymic	3.5(2–5)	4(2–4)	3(2–4)	0.682
Euphoric	3(2–4)	2(1–3)	2(1–4)	0.133

Categorical variables are described by frequency and percentage. Quantitative variables are described by mean and standard deviation for symmetric data or median (P25-P75) for asymmetric data. P for quantitative symmetric data determined by ANOVA followed by Tukey’s test, and asymmetric data by the Kruskal-Wallis test.

When patients who used current psychiatric medication were excluded (n = 95), the mean of smokers scored lower in the Control dimension than non-smokers did; (39.7±11.2 44.1±9.8; P = 0.032) even when adjusted for age and sex (P = 0.009; effect size: 0.16). Smokers scored higher on anxious temperament than ex-smokers (median 3[2–4] percentile; median 2[1–4] percentile; P = 0.007) and non-smokers (median 3[2–4] percentile; median 2[1–4] percentile; P = 0.005). According to the AFECT model, [26] people with anxious temperament are excessively worried, cautious, insecure, apprehensive, alert, vigilant and tend to avoid running risks.

An interesting finding is that in the subgroup BMI >50 Kg/m^2^, after having carried out adjustments for age and sex, we observed differences in emotional temperaments: ex-smokers scored higher on coping (P = 0.018) and control (P = 0.023) than smokers did (see Table 3). According to the AFECT model, [26] coping dimension regards how the individual faces adversities, how he or she is capable of finding solutions and learning with experience. Control involves monitoring the environment through attention, developing focus and planning strategies. [26]

**Table 3:** Characteristics of emotional and affective temperaments (AFECTS) of the subjects studied with BMI>50 kg/m^2^ (n = 103).

Characteristic	Smokers (n = 08)	Ex-smokers (n = 27)	No smokers (n = 68)	P
VOLITION	33.50±12.49	37.56±10.67	39.99±10.50	0.211
ANGER	32.63±7.57	28.41±12.22	27.97±10.01	0.497
INHIBITION	28.00±4.89	26.96±6.33	28.06±6.12	0.728
SENSIVITY	30.38±9.25	31.22±10.85	32.34±8.70	0.778
COPING	37.25±14.41	46.93±8.16	45.38±10.01	0.056*
CONTROL	36.13±11.96	44.48±8.64	42.88±10.06	0.113*
Depressive	2.5(1–3)	2(1–3)	1(1–3)	0.211
Anxious	3(3–4.75)	3(2–4)	2(1–4)	0.088
Apathetic	1.5(1–2)	1(1–2)	1(1–3)	0.838
Obsessive	3(1.25–4)	4(2–5)	3(2–5)	0.142
Cyclothymic	3(1.5–4)	2(1–4)	2(1–3)	0.183
Dysphoric	2.5(1.25–4)	3(1–3)	2(1–3)	0.271
Volatile	1.5(1–3.75)	1(1–3)	1(1–2)	0.258
Euthymic	2.5(1–3.75)	4(2–5)	4(2–5)	0.161
Irritable	4(3–5)	4(3–5)	3(3–4)	0.172
Disinhibited	1.5(1–4.75)	2(1–3)	2(1–4)	0.911
Hyperthymic	3(1.25–3.75)	3(2–4)	3(2–5)	0.601
Euphoric	2(1.25–3.75)	2(1–4)	3(1–4)	0.943

Categorical variables are described by frequency and percentage. Quantitative variables are described by mean and standard deviation for symmetric data or median (P25-P75) for asymmetric data. P for quantitative symmetric data determined by ANOVA followed by Tukey’s test, and asymmetric data by the Kruskal-Wallis test.

* adjusted for age and sex (multivariate linear regression): coping P = 0.023; control P = 0.018

## Discussion

Our study demonstrated an association between smoking status and affective and emotional temperaments, as measured by AFECTS, by analyzing obese patients undergoing the assessment process for bariatric surgery, excluding patients who were under psychiatric medication and with patients with BMI>50. These data had not been previously reported, and we are not aware of previous studies associating classes II and III obesity, smoking and temperament.

Amongst patients who were not using psychotropic drugs, smokers had lower scores on control and higher scores on anxious temperament compared to non-smokers, and these results were in agreement with Pompili et al., [41] who demonstrated that substance abusers had higher anxiety traits, impulsivity and suicide risk (less emotional control). These findings are clinically relevant because some specific types of temperament (depressive, cyclothymic, hyperthymic, irritable and anxious) can be considered subsyndromal (trait-related) manifestations and predispose to the development of mood disorders. [42] Moreover, in the groups of smokers with the risk of relapse to smoking after bariatric surgery, caution should be taken with the use of antidepressants in anti-smoking treatment, because they can cause mood instability, [43] and mainly with the anxious temperament that could predispose to suicidal behavior. [42] Both mood disorders and suicidal behavior are recurrent concerns of health care professionals working with bariatric surgery, because of their prevalence in this population. [44–46]

In the patients with a BMI >50 kg/m^2^, coping and control scores were lower in smokers than in ex-smokers and these temperaments characteristic may have contributed to the ability of ex-smokers to stop smoking.

An original contribution of this study was to demonstrate that temperament traits in class II and III obese bariatric surgery candidates did present differences in temperament, regarding smoking status. It was expected that the overlap of obesity and smoking would result in even more extreme temperaments, because both smoking and obesity have been shown in previous studies to be positively associated with novelty seeking, [13,15,16] higher anger, lower fear (inhibition), lower self-direction, [38] lower control and unstable affective temperaments. [14,17,47]

Even though we need more profound studies on the influence of temperament in obesity and tobacco usage the clinical importance of the present work is to demonstrate that through the perspective of temperament we may be dealing with subgroups of patients who are in need of specific adjustments to their temperament-based treatments. Likewise, tobacco usage in this population class II and III of obese patients must be observed in a broader spectrum during their psychiatric assessments. The use of tobacco should not be merely regarded by its harmful characteristics to the surgery intervention and recovery once it might represent the tip of the iceberg, on top of other psychiatric disorders and temperament characteristics that need to be addressed with particular attention aiming at a more qualified therapeutic adjustment in order to obtain better results in the middle and long term.

This study should be interpreted in the context of potential methodological limitations. Our sample had a high prevalence of Caucasians, and we do not know if we would find similar results in other ethnic groups. Furthermore, the temperament variable was assessed by a self-report questionnaire before evaluation for bariatric surgery and its padding may have been influenced by the patient's wish to be approved for operation. Also, we used self-reported smoking instead of laboratory testing. In addition, our results are restricted to obese people seeking treatment in a single service of bariatric surgery and provide no further information about the large number of obese people in the community or in other medical and non-medical places. Future studies for the validation of the AFECTS scale will be carried out amongst the population of obese people who fall in the categories of obesity classes II and III.

## Conclusion

Our study found out that the group of smokers presented a high score on anxious temperament as well as lower scores of Control than non-smokers when controlled for psychiatric medication. Smokers with BMI>50 presented lower scores on Coping and Control that ex-smokers did. These findings underscore the need for careful approaches on medical assessments in order to accomplish a wider comprehension of patients temperaments and clinical diagnoses and therefore maximize their treatment persistence and adherence, not only aiming at smoking cessation or at the prevention of smoking relapse, but also at other characteristics linked to control behavior such as dietary treatment, physical exercise and other related aspects. The external validity of these findings must be confirmed by others studies once our research was comprised of a very specific group of patients.
